# Misoprostol for the prevention of post-partum haemorrhage in Mozambique: an analysis of the interface between human rights, maternal health and development

**DOI:** 10.1186/s12914-020-00229-9

**Published:** 2020-04-08

**Authors:** Karen Hobday, Anthony B. Zwi, Caroline Homer, Renae Kirkham, Jennifer Hulme, Páscoa Zualo Wate, Ndola Prata

**Affiliations:** 1 Menzies School of Health Research, Charles Darwin University, PO Box 41096, Darwin, NT 0811 Australia; 2 Health, Rights and Development (HEARD@UNSW), Faculty of Arts and Social Sciences, University of New South Wales, Sydney, NSW 2052 Australia; 3 Burnet Institute, 85 Commercial Road, Melbourne, VIC 3004 Australia; 4 Department of Family and Community Medicine, University of Toronto, Toronto, Canada; 5 Department of Emergency Medicine, University Health Network, University of Toronto, Toronto, Canada; 6 Department of Women’s and Child Health, Ministry of Health, Avenida Eduardo Mondlane, Maputo, Mozambique; 7 Bixby Center for Population, Health and Sustainability, University of California–Berkeley, University Hall, Berkeley, CA 94720-6390 USA

**Keywords:** Post-partum haemorrhage, Maternal health, Human rights, Development, Mozambique, Right to health

## Abstract

**Background:**

Mozambique has high maternal mortality which is compounded by limited human resources for health, weak access to health services, and poor development indicators. In 2011, the Mozambique Ministry of Health (MoH) approved the distribution of misoprostol for the prevention of post-partum haemorrhage (PPH) at home births where oxytocin is not available. Misoprostol can be administered by a traditional birth attendant or self-administered. The objective of this paper is to examine, through applying a human rights lens, the broader contextual, policy and institutional issues that have influenced and impacted the early implementation of misoprostol for the prevention of PPH. We explore the utility of rights-based framework to inform this particular program, with implications for sexual and reproductive health programs more broadly.

**Methods:**

A human rights, health and development framework was used to analyse the early expansion phase of the scale-up of Mozambique’s misoprostol program in two provinces. A policy document review was undertaken to contextualize the human rights, health and development setting in Mozambique. Qualitative primary data from a program evaluation of misoprostol for the prevention of PPH was then analysed using a human rights lens; these results are presented alongside three examples where rights are constrained.

**Results:**

Structural and institutional challenges exacerbated gaps in the misoprostol program, and sexual and reproductive health more generally. While enshrined in the constitution and within health policy documents, human rights were not fully met and many individuals in the study were unaware of their rights. Lack of information about the purpose of misoprostol and how to access the medication contributed to power imbalances between the state, health care workers and beneficiaries. The accessibility of misoprostol was further limited due to dynamics of power and control.

**Conclusions:**

Applying a rights-based approach to the Mozambican misoprostol program is helpful in contextualising and informing the practical changes needed to improve access to misoprostol as an essential medicine, and in turn, preventing PPH. This study adds to the evidence of the interconnection between human rights, health and development and the importance of integrating the concepts to ensure women’s rights are prioritized within health service delivery.

## Background

In 2015, approximately 303,000 women worldwide died during pregnancy and/or due to childbirth complications and almost all in low to middle income countries [1]. The main causes were post-partum haemorrhage (PPH), sepsis, pre-eclampsia or eclampsia, complications arising at the time of birth and unsafe abortion. All of these causes are, to a large extent, preventable [1]. PPH alone accounts for nearly one quarter of all maternal deaths worldwide [2].

Oxytocin (administered intravenously or intramuscularly) is recommended for the prevention of PPH, but when oxytocin is not available, misoprostol, a medication available in tablet form (600 mcg oral) can be used as an alternative [3]. Oxytocin is rarely accessible to women giving birth outside a health care setting due to the need for refrigeration and administration via injection [4, 5]. Oxytocin is widely used in health facilities across Mozambique but 30% of births take place without a skilled birth attendant (SBA) and therefore many women give birth without uterotonic protection (IMASIDA, [6]).

Misoprostol is a safe alternative for the prevention of PPH when administered immediately after the birth where oxytocin is not available [3]. Misoprostol is available internationally in 200mcg tablets. Therapeutic or preventive doses for PPH are usually administered as 3 tablets (600mcg) or 4 tablets (800 mcg) [3]. Misoprostol can be self-administered or administered by a trained community health worker (CHW) or traditional birth attendant (TBA) [7, 8].

Mozambique has a population of 28.9 million with an average life expectancy of 55.1 years [9, 10]. In 2015, Mozambique’s maternal mortality ratio was estimated at 489 per 100,000 live births [11]. Haemorrhage, both antepartum and post-partum, is a leading cause of maternal deaths [12–14].

In 2009–2010, the Ministry of Health (MoH) commissioned a study to assess the effectiveness and feasibility of distributing misoprostol for the prevention of PPH in the community. The study showed that distribution through both TBAs and antenatal care (ANC) achieved 99% misoprostol use among women who gave birth unassisted at home [15]. Subsequently, the results supported the MoH to plan for the distribution of misoprostol for PPH prevention at the community level via ANC and TBAs.

In 2015, the National Strategy for the Prevention of PPH at the Community Level (referred to as the National Strategy for PPH) was launched by the MoH and partners. The strategy outlined a plan to roll-out the misoprostol program in 35 of 128 districts across the country. Since then, the MoH and a number of MNCH partners have contributed technical support for the implementation of the misoprostol program including Jhpiego’s Maternal and Child Survival Program (MCSP) and the United Nations Population Fund (UNFPA).

Human rights and gender equality are well enshrined in legal frameworks in Mozambique, including the first Constitution in 1975 [16]. Mozambique is signatory to most major human rights treaties aimed at promoting equal rights for women and men (See Additional file 1). However, in practice a number of barriers prevent women from accessing their rights, including access to misoprostol.

Human rights based approaches can be used to ensure rights are embedded into a program or policy from the onset and incorporated into each step of the program cycle including policy, planning, implementation and monitoring and evaluation [17]. The essential components of a human rights approach include: universality with a focus on vulnerable populations; indivisibility/interdependence; accountability; participation; rule of law; and progressive realization, access, affordability, quality [18, 19].

**Fig. 1:**
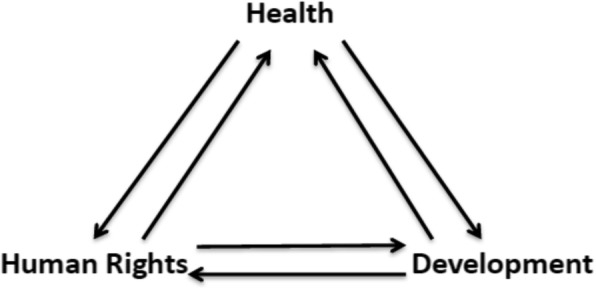
The Health, Human Rights and Development Triangle (Originally printed in Tarantola et al., 2008 [20] with permission obtained to re-print. This figure has been republished in Tarantola et al., 2013 [21])

Health, human rights and development interface in a simple framework (Fig. 1) designed by Tarantola et al. [20, 21] that can be used to analyse a state’s capacity to uphold human rights, provide essential health services and encourage human development. The framework goes beyond the usual programmatic challenges by forcing more in-depth, nuanced understanding of relevant systems [20, 21].

This paper applies a rights-based framework to understanding the broader contextual, policy and institutional issues at play in the early implementation of misoprostol for the prevention of PPH. We then explore the utility of a rights-based framework to inform the Mozambique misoprostol program as a unique case study, and consider the implications for sexual and reproductive health programs more broadly.

## Methods

This paper presents an analysis of the early expansion phase of the scale-up of Mozambique’s misoprostol program in two provinces through the lens of a Tarantola and colleagues’ human rights, health and development framework [20, 21] (see Fig. 1). This qualitative study had two main components. The first was a policy document review to describe the human rights, health, and development context in Mozambique. The second was a thematic analysis of qualitative primary data from a program evaluation of misoprostol for the prevention of PPH. The health, human rights and development framework was used to identify three examples from the primary data which describes where rights might have been infringed.

### Policy document review

A document review of misoprostol program and policy documentation in Mozambique was undertaken with the goal of identifying the key documents most salient to health and reproductive rights. These documents were initially accessed through a grey literature search online, using a combination of key words in English and Portuguese including “health”, “development” AND/OR “human rights” in “Mozambique”. We then triangulated our grey literature search with the MoH and program partners through the course of the study. Core documents included the National PPH Strategy; The National Health Sector Strategic Plan 2015–2019; the National Plan for Health and Human Resource Development; Demographic and Health Surveys (2011 & 2015); the international human rights treaties Mozambique has signed and ratified; the UNDP Human Development Index; and gender equity publications and policy areas related to health and development at the national, regional and organisational levels. Forward snowballing of references and suggestions from the MoH and program partners were used to locate additional documents and literature. A thematic analysis of the documents was undertaken under the Framework’s central tenents of human rights, health and development [20].

### Analysis of qualitative primary data

Qualitative data from semi-structured interviews and focus group discussions were collected in 2017 as part of a larger evaluation of the scale-up of misoprostol for the prevention of PPH in two provinces in Mozambique [22]. The broader study aimed to a) uderstand TBA’s roles and perceptions on the distribution of misoprostol; b) explore the views of women who had used misoprostol; c) identify facilitators and barriers to the early expansion of the misoprostol program for the prevention of PPH at the community level; and d) examine coverage and utilisation of misoprostol in the two provinces.

Study interview guides for stakeholders and health staff were developed using existing tools relevant to this study [23, 24]. Questions focussed on the process of scale-up including barriers and enablers; dissemination and advocacy; organizational inputs; finances/mobilization of resources; and monitoring and evaluation. CHWs and TBAs were interviewed about their use and understanding of misoprostol and the barriers and enablers to the misoprostol program. Women who used misoprostol were asked questions about their experience receiving and using the medication.

Purposive sampling was used to select the research participants based on consultation with key stakeholders in the program, assistance from district health staff, community health workers, and women who used misoprostol. MNCH stakeholders were interviewed based on their involvement in the misoprostol program. Details of recruitment and data collection procedures are documented elsewhere [22, 25].

In total, focus group discussions and semi-structured key informant interviews were undertaken with:

i. 15 Ministry of Health staff and policy/MNCH stakeholders.
ii. 19 health staff involved in the misoprostol program (Medical directors, hospital directors, MNCH nurses, midwives, health technicians/auxiliary staff and pharmacists).
iii. 15 community health workers and coordinators, 15 traditional birth attendants (TBAs) and 11 women who had used misoprostol.
iv. 4 focus groups discussions (FGDs) with TBAs in Nampula Province.

Most interviews and FGDs were conducted in local language or where appropriate, Portuguese. Interviews averaged 45 min and were recorded with participant permission. MNCH stakeholder interviews took place in Maputo city, Inhambane and Nampula Provinces at the participant’s office or convenient alternative. Team members debriefed daily to record observations and context. Recruitment and data collection procedures are elaborated elsewhere [22, 25].

Two international and three local research assistants collected data. Research assistants received training on qualitative data collection and research ethics. Interviews, field notes and recordings were translated and transcribed in Portuguese by Mozambicans who spoke local dialects, and then translated into English. Data quality checks from Portuguese to English were undertaken by KH and JH by listening to the interview recording in Portuguese alongside the Portuguese transcripts and English translation. Nvivo (v.11) software was used to undertake the analysis. The first author read all interviews and documents twice to become familiar with the data. All data were deductively coded by the first author according to the underpinning constructs of the framework [20]. The second round of coding was inductive and resulted in the emergence of additional themes. The analysis was cross-checked with co-authors to ensure interpretation of meaning was accurate.

Ethical clearance was obtained from the Human Research Ethics Committee at Charles Darwin University, Australia (HREC 2015–2445) and the Mozambican National Bioethics Committee and MoH (105/CNBS/2016). All participants were informed of the study purpose, potential risks and benefits, after which written informed consent was obtained. All participants were given the right to refuse or withdraw at any point; none did so.

## Results

We first present the broad health and policy context in Mozambique that emerged from the policy document analysis. An overview of the misoprostol program follows from both primary and secondary data sources. Finally, three case studies, or situations, were chosen from the qualitative data to highlight how a rights-based approach could assist women, TBAs and health staff to access their rights and improve program delivery.

All data were deductively coded into the three categories of the framework; human rights, health or development. A second round of coding was undertaken in each category resulting in additional themes. Themes that emerged within the human rights category included, UN Conventions, Mozambican women’s rights; access to information; punitive/threatening behaviour; and abortion. Under the health category, themes included access and quality of maternal health care; pressure to increase health facility deliveries; and health systems. Themes in the development category included economic situation; Sustainable Development Goals; gender equity; distance and infrastructure.

### Human rights, health, and development context

#### Human rights

The human rights domain reflects the international, regional and national laws and policies that pertain to a state’s legal system. The results presented here include UN conventions that have been ratified and endorsed, the status of Mozambican women’s rights, abortion laws, access to sexual and reproductive health (SRH) information, and limitations on the distribution of misoprostol due to the eligibility criteria within the program.

Mozambique is a signatory to major international human rights conventions which recognize the human right to health. For example, Article 12 of the Convention on the Elimination of All Forms of Discrimination Against Women (CEDAW) makes specific mention of reproductive health rights; states have the responsibility to ensure that men and women have access to health services including family planning and women receive free pregnancy, post-natal, labour and birth services, and information to decide on birth spacing [26]. Other human rights instruments to which Mozambique is signatory can further advance reproductive and sexual health (See Table 1 in Additional file 1). Mozambique is not party to the International Covenant on Economic, Social and Cultural Rights (ICESCR) which contains Article 12 and General Comment 14, the right to the highest attainable standard of health [29]. State authorities have cited a lack of resources to uphold the rights within the ICESCR as the predominant reason for abstaining from signing the covenant [30].

**Table 1:** Maternal and child health indicators in Mozambique

Indicator		Source, Reference
Maternal Mortality Ratio	489/100000	(World Health Organization, 2015) [11]
% Births attended by a Skilled Birth Attendant	70 (91% urban; 67% rural)	(IMASIDA, 2016) [6]
Lifetime Risk of Maternal Mortality	1/40	(World Health Organization, 2015) [11]
Neonatal Mortality Rate	27.1/1000 live births	(World Health Organization, 2016) [27]
Fertility Rate (ages 15–49; 2015)	5.9 (6.6 rural; 4.5 urban)	(IMASIDA, 2016) [6]
Demand for family planning for 15–49 year olds	50% (47% rural; 57% urban)	(IMASIDA, 2016) [6]
Access to contraception for 15–19 year olds	14.1%	(Family Planning 2020, 2017) [28]
% of women > 25 with some secondary education in 2015	2.8%	(IMASIDA, 2016) [6]
% of men with some secondary education in 2015	8%	(IMASIDA, 2016) [6]

The Mozambican government, including the MoH, refers to the human right to health within key national documents including the National Health Sector Strategy 2014–2019 which states;

*The vision, mission and guiding principles […] were developed based on the premise that health constitutes a basic human right, as such, they reflect the long-term values and aspirations of Mozambicans. This approach aims to substantiate the right to health and other related human rights reflected in the country’s legal framework* [31].

Access to contraception and the ability to negotiate contraceptive use and plan pregnancies are reproductive health rights as stated in Article 12 of CEDAW [26]. Increasing access to sexual and reproductive health services, especially modern contraception, is a main objective in the 2014–2019 Health Sector Strategy [31].

In 2014, the abortion laws in Mozambique were reformed, which increased legal access to safe abortion [32]. Women could now access a safe abortion up to 12, 16 or 24 weeks depending on their circumstances [32]. All abortions must be provided in a health facility with a qualified health professional. Parental consent must be obtained for girls under 16 years of age or those with mental illness [33]. In 2016–2017, the Ministry of Health and partner organizations developed clinical guidelines for abortion and post-abortion care [33]. At the time of data collection, abortion remained accessible only at some tertiary and quaternary hospitals and was difficult to access in rural areas [34].

### Health

The health domain includes an analysis of key health and health system indicators. This section examines Mozambique’s maternal health indicators, human resources for health, and health supplies as a measure of the country’s progress in upholding the right to health (See Table 1). Maternal health is a good indicator of women’s status in terms of health, development and human rights. Health and development are directly connected; enjoyment of the right to health is a prerequisite for, as well as an outcome of, development [21].

High maternal mortality and morbidity persist in Mozambique. Between 1990 and 2013, the country experienced a 64% reduction in maternal mortality [35]. Despite this, Mozambique did not meet Millennium Development Goal (MDG) 5 to reduce the maternal mortality ratio (MMR) to 330 per 1,000,000 [36]; the MMR remains extremely high (489/100000 live births) [11]. The lifetime risk of maternal death remains high; one in 40 Mozambican women will die during childbirth [11].

Mozambique has a high fertility rate (5.9), and limited access to family planning. Nampula Province has a higher fertility rate at 6.1 versus Inhambane Province at 4.9 (IMASIDA, [6]). In 2017, only 14.1% of youth between the ages of 15–19 had access to contraception [28]. Abortion is a leading direct cause of maternal mortality in Mozambique [31]. The 2011 Demographic Health Survey (DHS) revealed that 9% of women aged 15–49 years reported that they had ever terminated a pregnancy [37].

The MoH identified the reduction of maternal and neonatal mortality as the first of five key national health priorities outlined in the National Health Sector Strategy 2014–2019. One of the main objectives to reach this target was to expand and improve health services. The second strategic goal under this objective was related to misoprostol; ‘…and introducing and expanding the use of Misoprostol at HF [Health Facility] and community level for the prevention and control of post-partum haemorrhage’ [31].

#### Health services

Maternal health services in Mozambique are well described elsewhere [31]. The country has made significant strides in increasing the number of qualified health staff since the end of the civil war [38]. However, the ratio of qualified heath providers to patients remain insufficient; in 2013 there were 0.05 physicians and 0.40 nurses and midwives per 1000 population [27]. There are vast disparities between rural and urban populations in terms of access to reproductive health services, quality of care, transport and infrastructure. Rural women have less access to health services and skilled birth attendants (67% rural versus 91% urban) than those living in urban locations [6].

Essential medicines in Mozambique are distributed through the Centre for Medicines and Drug Supplies in Maputo and a regional warehouse located in Beira, Sofala Province. Medications are sent to 10 provincial warehouses and central hospitals across the country and sent to the district level warehouse for distribution to health facilities. The MoH supply chain experiences weaknesses in distribution from district to health facility level. Between 2011 and 2013, 85% of 13 district warehouses in Sofala Province experienced a stock-out of an essential drug and all had a stock-out of an essential product at least once in the three annual stock assessments [39]. Medicine stock-outs at the health facility level are most strongly associated with distance from the district warehouse [39]. In 2015, a UNFPA assessment found that 84% of health facilities across Mozambique had at least seven essential maternal and reproductive health medicines, an improvement from previous years (59% in 2014) [40].

Only half of rural health facilities have electricity or solar power systems and 60% have a water supply [31]. In 2015, 11% of Mozambican health facilities lacked access to a cold chain to conserve medical supplies, including oxytocin [40]. Poor infrastructure, lack of transportation, limited numbers of health facilities, and quality of services all impact access to rural health services in Mozambique [41, 42].

### Development

The development domain examines the processes and goals of human development which improve people’s opportunities and ultimately their lives [21]. Development was recognized as a right in 1986. Several development issues facilitated the expansion of the misoprostol program and the health and human rights of women. Those included Mozambique’s key economic indicators, progress on Sustainable Development Goals (SDGs), gender equity, literacy rates and access to education.

In 2015, Mozambique ranked 182 out of 187 countries in the UNDP Human Development Index, making it one of the least developed countries in the world [43]. Gross National Income per capita is $1098 [43]. Overall, Mozambique ranked 29 of 51 countries in the 2018 African SDG report (The SDG Center for Africa and Sustainable Development Solutions Network, 44). The country is experiencing serious challenges attaining nine of seventeen SDGs, including poverty, hunger, health and well-being, education, gender equality, water and sanitation and innovation, industry and infrastructure [44]. The country ranks particularly low in the first Goal to end poverty, with 66.6% of the population living on less than 1.90 USD per day, and the second Goal to end hunger, with 26.6% of the population undernourished and 41.2% stunting among children under the age of five [44]. While the net primary school enrolment is high at 89.6%, on average children spend only 3.5 years in school.

Health financing directly impacted progress of the misoprostol program. In 2017, the health sector received US$ 300.1 million which was 7.8% of the total value of the State Budget [45]. State funding for general health operations and human resources was largely seen as being insufficient. The budget for the misoprostol program was largely dependent on external donor funding and was not fully realised. This impacted the program as monitoring and evaluation including supervisory visits was largely unfunded.

In 2017, Mozambique ranked 139 out of 159 countries in the Gender Inequality Index [46] with particularly poor ranking in reproductive health and access to education. The Global Gender Gap report ranked Mozambique 130 of 144 countries in terms of women’s educational attainment. Women have a lower literacy rate than men (36.5% versus 67.4%) and lower enrolment at primary, secondary and tertiary schools [47]. Only 2.8% of women over the age of 25 had some secondary education versus 8% of men (IMASIDA, [6]). Female representation in parliament was fairly high (39.6%) as was involvement in the workplace (82.5%) [43].

Although women have high participation in the labour market, they predominately work in poorly paid work including subsistence farming and domestic labour and often do not have rights to the land they farm or the income they earn [48, 49]. Women earn 80% of the wage that a man does for the same work [50]. The application of gender policy and women’s rights is weak given the dominant patriarchal socio-cultural order, limited implementation and a passive judiciary [16, 51].

### Misoprostol program

The National PPH Strategy outlines the misoprostol program to reduce the incidence of PPH with a plan for universal distribution to women [14]. In 2015, the program was initiated in 6 districts in Sofala and Inhambane Provinces. The intention was to learn from these sites and adapt the program as necessary in the remaining 29 districts [52].

**Fig. 2:**
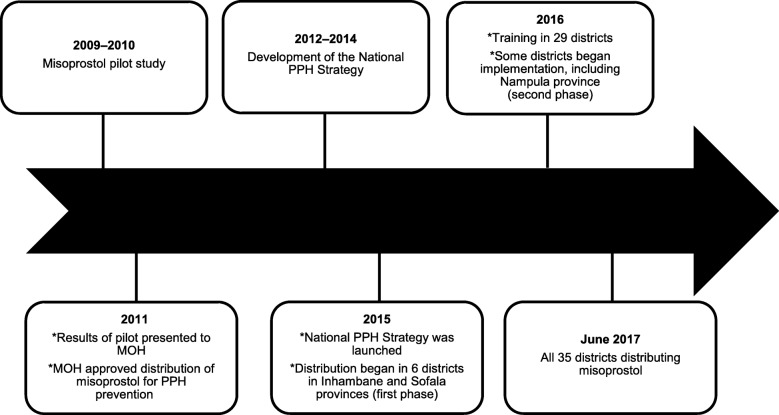
The Evolution of the Distribution of Misoprostol for Prevention of Postpartum Hemorrhage in Mozambique (Figure developed by K. Hobday and was published in Hobday et al., 2019 [22] which is an open access journal)

As of 2017, misoprostol for the prevention of PPH has been introduced in 35 districts and is led by the MoH at Central, Provincial and District levels [22] Misoprostol is on the Essential Medicines List (EML), and is distributed via the MoH pharmaceutical system. UNFPA funds the procurement of misoprostol and initial training of health staff, CHWs and TBAs. Jhpiego’s MCSP Program funded training and the provision of technical support in two provinces. The MoH depends on external funding for the program and the 2.6 million USD budget for the misoprostol program was not fully funded [22] See Fig. 2 for a historical overview of the program.

MNCH nurses and midwives were trained to distribute misoprostol to women in advance at antenatal care appointments after 28 weeks gestation. However, not all MNCH staff received training; approximately a quarter of the MNCH nurses interviewed said they had not received formal training in the program. Some MNCH nurses reported some discomfort distributing the medication which was a barrier to implementation [22]. Other health staff, including pharmacists, did not receive training. Interviews revealed that there was limited dissemination of the National PPH Strategy, particularly at the district level and many pharmacists were unaware of the strategy. TBAs were trained to distribute misoprostol directly to the woman after she gave birth and before the placenta was born. TBAs received the dose of misoprostol (3 pills) from their local CHW.

In 2015, soon after the program began, the MoH introduced selection criteria to increase controls to reduce the risk of misuse for abortion and limit the amount of misoprostol in the community. The criteria outlined which women can qualify for advanced distribution of misoprostol at the health facility. The criteria stated that a pregnant woman must be registered for antenatal care where misoprostol is available and have reached 28 weeks gestation. She must additionally meet at least one of the following criteria: 1) a history of giving birth at home/outside of the health facility; 2) reside more than 8 km from a health facility; 3) be a grand multipara (more than 5 previous births); 4) a current or past history of multiple pregnancies; 5) a history of postpartum haemorrhage.

Disparities were found between the two provinces in terms of access and utilisation of misoprostol. Nampula Province reported very high coverage whereas Inhambane reported lower coverage. Higher coverage in Nampula was possibly due to encouragement from political champions, support from Jhpiego’s MCSP program and previous knowledge of the benefits of misoprostol due to the 2009–2010 pilot study that took place in the Province. Interviews in Inhambane found that low coverage was in part due to the strong priority health staff placed on applying the selection criteria, medical directors and pharmacist’s perceptions that misoprostol may be misused for abortion or used incorrectly and the emphasis of the MoH on encouraging facility births. Further details of the access and utilisation are presented elsewhere [22].

The MoH leads the misoprostol program and has begun to institutionalise it within the health system in the 35 target districts. However, our previous study found a number of barriers surrounding coverage due to the adaptation from universal to select distribution; fear of misdirecting misoprostol for abortion or labour induction; limited dissemination of the National Prevention of PPH Strategy; insufficient monitoring and evaluation; challenges with the logistics system and an inability to engage remote Traditional Birth Attendants [22]. Stakeholders from a number of districts reported shortages of misoprostol stock, despite submitting requests to the provincial pharmacy. The lack of a regular supply of stock appeared to be a major reason for low coverage in Inhambane Province. Although the program was led by the MoH, it is currently taking place in only 35 of 128 districts and some health staff questioned the sustainability of the program.

### The infringement of rights: three examples

This section of the paper presents three examples derived from the analysis of primary data to illustrate where rights were infringed. The first example describes women’s experiences of discrimination for not giving birth in a health facility. The second example surrounds access to information about misoprostol. The third depicts issues of power and control in cases of who is permitted to receive and provide misoprostol for the prevention of PPH. Each of these examples illustrates gaps in current practice, and to demonstrate the added value of integrating a rights-based framework into health care system development.

#### Unintended consequences: emphasis on facility deliveries

According to the National PPH Strategy, districts involved in the scale-up should have a maternal waiting home attached to the health facility. In practice, only half of the health facilities in the study had maternal waiting homes, most of which were unfinished or not in use. Even in districts where maternity homes were available at the health facility, some women explained that due to a lack of transportation, money, child care and support from their husband they felt they had no choice but to wait until labour had started before trying to reach the health facility:

*Usually even the women realizing the expected date of delivery will not go to the health unit for fear of the long waiting time and therefore, do not want to leave the smaller children alone at home. Men are not staying very long with children; others are mothers whose husbands are working in South Africa or are single. (TBA 13).*

In some cases TBAs urged women to walk for kilometres to the health facility to give birth. As a result, women reported that they gave birth on the side of the road which put them at risk of exposure and infection. All of the women who took part in the FGD in Nampula Province said that they had walked in labour to reach the health facility. TBAs described the challenges of accompanying women walking to the clinic, some walking for hours slowly and in excruciating pain. One TBA described it as ‘crawling’ as in this quote:

*You know what it means to walk for a woman with contractions, it’s practically crawling and she says “I hurt a lot, I can not walk” and we sensitize her to be strong and try walking and then when I see that she is on the edge I say, “Stop to not compromise the well-being of the newborn” (TBA 16).*

All of the women who took part in the FGD in Nampula Province said that they had walked in labour to reach the health facility. This highlights the challenge of accessing transportation and/or money to travel to the health facility in a timely fashion, increasing risks of giving birth outdoors, in unhygienic conditions and/or without a SBA.

Some women and TBAs said that they feared punishment from health staff for births that took place at home. Some women were even denied access to the newborn’s immunization cards or were charged a fee to obtain them:

*We tend to be afraid to leave the woman without help because… to get a record [birth registration record], it gets complicated, as if we midwives [TBAs] we were encouraging them not to come to the hospital to give birth… They suffer because they are not given the files because they gave birth outside the maternity ward (TBA 18).*

Women in one of the provinces who used misoprostol and gave birth in the community revealed that they had all bought the newborn’s immunization cards at varying costs (50–100 meticais equivalent to .80–1.60 USD in 2018). One woman said, “*I paid 100 meticais. That does not have a fixed price ... they decide the amount they want to charge ... is this a legal procedure?” (Woman who used misoprostol, Nampula).*

While many women stated that they preferred to give birth at a health facility, some TBAs and women reported that the fear of being reprimanded and denied the newborn’s consultation card by health staff was the main factor for attempting to reach the health facility, even if labour was in progress.

#### Limiting the right to receive, request and provide information about misoprostol

The second example depicts the lack of information provided to the community about the benefits of misoprostol, and how to access and use it. In some locations, women said there was limited information about misoprostol, resulting in fear and misinformation. Women in a focus group highlighted this concern saying:

*People who have not yet taken it [misoprostol] say they are afraid to take it because they do not know what the reaction will be. Now we have tried to pass on to them our experience that it is a good medicine and that it does not harm the body, that it does not kill, we are here alive and healthy (FGD 6, Erati, Nampula).*

Some women who had used misoprostol reported that men in the community were suspicious of the medication and did not want their wives to use it. For example, ‘*The men in the villages say that medicine [misoprostol] is not good. They tell women not to go back and not to take it (Woman who used misoprostol, Nampula).*

Other women however said their husbands were supportive:

*They [husbands] praise [misoprostol] a lot because they know full well that the woman who loses a lot of blood during birth has many consequences. For example, when she lost a lot of blood she gets to the point of not being able to lift and carry a simple can of water; this suffering can last about two or even three months…It is different when the woman uses this medicine, she gains strength and has good physical appearance (Woman who used misoprostol, Inhambane).*

Some stakeholders believed that misoprostol for the prevention of PPH was not yet widely known to be available amongst health staff and the community due to the limited geographic reach of the program. One stakeholder stated that, *‘Once more women know about the misol [misoprostol] and understand it is their human right to have it, they will be more demanding’ (Stakeholder, Maputo).* Many stakeholders felt that more work was needed to further disseminate the National PPH Policy across the country.

#### Effects of power and control to limit access to medication

The final example describes issues of power and control over which women were chosen to access misoprostol in advance at ANC appointments, and how much agency was given to the TBAs to provide safe birth services. The introduction of selection criteria to further control the circulation of misoprostol meant that women who delivered at home were excluded from receiving advanced misoprostol. Some health staff supported this decision as they believed misoprostol should be carefully controlled. For example:

*People know that this [misoprostol] is for abortion. It will be used two-fold and you need to be aware of it. Post-partum haemorrhage is a big problem we have to deal with but you also need to know that the TBAs need to receive the correct training and the APEs [CHWs] as well. Well misoprostol can be helpful but it can be dangerous as well (Stakeholder, Maputo).*

However, other health staff believed that the criteria significantly reduced the number of women able to receive protection from an uterotonic. One stakeholder felt that use of the selection criteria discriminated against women and depended on the nurse or midwives personal judgement of who can walk while in labour to the health facility, stating, *“Who can tell out of ten women who will have a PPH?” (Stakeholder, Maputo).*

Interviews revealed that some health staff limited their distribution of misoprostol due to their personal values about abortion. Some health staff limited their distribution of misoprostol due to their perceptions and biases surrounding the potential for CHWs, TBAs and women to misuse misoprostol intended for prevention of PPH for abortion. They were concerned that a medication which was previously highly restricted was now permitted for distribution at antenatal care to women in advance of their labour and to TBAs. One health staff stated:

*The misoprostol was a controlled drug and is even indicated for abortion. Because we have this on our conscience, we keep guarding it. It is not to distribute to any health unit or person we find looking for it (Health staff, Inhambane).*

TBAs spoke of the challenges they experienced working with the misoprostol program with limited resources. TBAs receive chlorhexidine and misoprostol from the district health centre or health post but do not receive clean birth kits to help facilitate their work. TBAs worried about the risk of infection to themselves, the women and the newborns. The majority of TBAs expressed concern about assisting women to give birth at home without gloves, water or soap, for instance:

*It’s about the gloves, we only have them here in the health unit, we do not have them at home. We only use plastic [to cover their hands] and in these times of illness we take risks during childbirth, so we are worried (TBA 6).*

In each of the three examples above, incorporating a rights-based approach would shed light on a range of other interventions and program improvements which have not, to date, received attention.

## Discussion

This article presents a case study of the Mozambique MoH-led program to distribute misoprostol at the community level to prevent PPH. We applied a human rights, health and development framework to understand the broader contextual, policy and institutional issues that may have influenced and impacted the misoprostol program. Results from this study and the description of the three examples where rights might have been infringed, highlight some of the areas warranting further attention to improve Mozambican women’s access to misoprostol for the prevention of PPH and SRH more generally. The broad policy objectives of the National Health Sector Strategic Plan and National PPH Strategy are laudable; however the reality of what occurs in practice must be urgently considered.

Similar to many low income countries, health care often fails at the point of delivery – despite laudable policy commitments [53]. The right to health is enshrined in the many national laws and regional and international treaties which Mozambique is party. The state has an obligation to fulfil the rights of its citizens including to protect and promote women’s reproductive rights [54, 55]. The human right to health is at the core of the existing National Health Sector Strategic Plan and the development of the National PPH Strategy was a positive step to elevating the need to reduce PPH. However, with constrained funding and capacity, the state in turn claims that it is unable to uphold the right to health and its core elements including quality, access, availability and affordability. This is particularly true for SRH: Cook states, “where they [SRH laws] exist, they are rarely or inadequately enforced” [56].

A rights-based approach offers a framework to advance human development through and accompanied by the promotion and protection of human rights [18]. The approach suggests that all programs intentionally guide and support capacity building of duty bearers to meet their obligations to international human rights [20]. The foundation of the human rights-based approach is the obligation of duty bearers, notably states but also those providing services, to deliver rights. How could these principles and policies practically translate into health justice? How might we apply a human rights-based approach to the misoprostol program to improve accessibility, affordability, quality and non-discrimination?

Institutions and organisations using this approach must provide relevant and transparent information to their beneficiaries [57]. The MoH would shift focus to communities to provide accurate information about how and when to use misoprostol, potential side effects and are made aware of their SRH rights. Not all health staff were aware of the National PPH Strategy which may have impacted the low distribution of misoprostol in the study locations. The application of a rights-based approach would bolster efforts to share the National PPH Strategy with health staff and communities alike.

Health staff should facilitate active participation and engagement of the community in all stages of the program from design to implementation. Participation ensures that services are appropriate and encourages rights-holders to exert agency to claim their rights [58]. In addition, partners can encourage the MoH as duty-bearers to respect SRH rights. Women should be repositioned to be at the centre of women’s health program planning, monitoring and evaluation.

The SDG goal to reduce MMR is supported by encouraging facility-based births with an SBA. However, the example of strict encouragement of facility-based birth highlighted the limited recognition of unintended negative consequences. There is clearly a need to consider the challenges women experience in reaching facilities in the presence of limited resources or a lack of transportation. Strategies directed at increasing facility births are expected in all maternal health programs; combining this with protection and care of individual women who cannot for social and economic constraints access a health facility is not disingenuous or sending “mixed messages”, but rather upholding women’s SRH rights as the country invests in infrastructure, builds trust and improves the quality of services.

Evidence of giving birth while walking to the facility violates women’s rights to quality health care and privacy and ignores her safety and dignity. Improving access to emergency transportation to health facilities in remote locations is central to improving maternal health outcomes. The MoH may consider global best practices to secure funding and systems to increase the number of ambulances, including tricycle or motorcycle ambulances; increasing outreach services; and work with communities to develop community savings schemes or other forms of pooled funds to assure emergency transportation.

Discrimination, punishment and extortion for women who are unable to reach the health facility to deliver violates the right to birth registration. This is - a fundamental human right stated in article 24, paragraph 2 of the International Covenant on Civil and Political Rights and article 7 of the Convention on the Rights of the Child [59]. Without the newborn’s immunization cards, families are unable to register the birth of their child if born outside of a hospital. This institutional barrier violates both the mother’s and child’s rights and reflects major imbalances in power and control. A similar phenomenon was reported in Sierra Leone, where patients were often required to pay for health services, including their child’s immunization record, despite stipulation that healthcare is free [60]. Women disproportionately suffer the consequences of additional out of pocket expenditures [57]. Increasing national expenditure on health and ensuring health staff are fairly compensated and have adequate resources to conduct their work would likely reduce corruption, in combination with efforts to bolster social accountability. The MoH can reiterate with health staff that their duty is to issue consultation/birth registration cards to all newborns, regardless of location of birth; the public in turn must know it is their right to register their babies without paying bribes, and have recourse to complaint systems where their rights are not met.

Examples of access to limited information highlight the need for proactive efforts to ensure information about SRH services, facilities and how to access them. The right to receive, request and provide information is protected by all human rights conventions [56]. The lack of information may have influenced men’s negative perceptions about misoprostol, leading to reduced uptake by women.

Feinglass, Gomess & Maru have worked to support a cadre of grassroots health advocates in Mozambique that blend legal empowerment and social accountability: they raise awareness on health policy and people’s rights, help clients seek justice for their complaints, and facilitate dialogues between communities and health staff [61]. Their positive experiences addressing over a thousand grievances across 27 health facilities suggests that when people know their rights and are equipped to exercise them, the health system improves. Women and communities first need to know of the purpose of misoprostol and where it is available.

The final example depicted the dynamics of power and control between who was permitted to receive misoprostol. This revealed that the application of the selection criteria limited the number of women targeted to receive advanced distribution of misoprostol for the prevention of PPH. MNCH nurses restricted access by strictly applying the criteria and in some cases using their personal judgement and biases to decide who should receive misoprostol. The right to essential medicines, as defined under the WHO Action Programme on Essential Drugs, is also a core obligation of the state [19]. The Mozambican government is obliged to uphold this right and ensure all citizens have access to essential medicines, including misoprostol. Since 2002, numerous studies have been undertaken on the feasibility of distributing misoprostol for the prevention of PPH at the community level. The studies were conducted in low income countries and revealed that the vast majority of women were able to correctly administer misoprostol themselves or with CHW assistance [62]. It will take time to shift the culture to trust CHWs to distribute misoprostol and women themselves to safely use it. A rights-based approach will re-frame the issue to reinforce that women have a right to access this life-saving medication – whether this is through CHWs, TBAs or advance distribution to the women themselves.

## Limitations

The research was undertaken in two provinces of Mozambique and cannot be widely generalised. However, the case study highlighted issues that warrant systematic rights-based responses; these are likely to be applicable elsewhere. Secondary data sources formed the basis of the analysis on human rights and development; further primary research on these issues would be of value.

## Conclusion

The human rights, health and development framework revealed structural and institutional challenges which exacerbated gaps to the misoprostol program. Mozambique has a strong foundation for supporting human rights, including the right to sexual and reproductive health. The MoH has prioritized the reduction of MMR within the five key priorities of the National Health Sector Strategy 2014–2019. In addition, the 2014 abortion reform was a positive step towards improving access to sexual and reproductive health rights. However, Mozambique has not ratified the ICESCR which is the most important Covenant for the right to health. Human rights are not fully upheld by the state and more importantly many citizens are unaware of their rights, including their right to SRH.

Through the misoprostol program, Mozambique has made strides in improving uterotonic coverage to women who are unable to reach a health facility to give birth. Applying a rights- based approach to the implementation of the misoprostol program combined with improvements to ensure health services are accessible, available, affordable and of high quality are needed to improve maternal health outcomes in the country.

From our knowledge, this is the first application of the health, human rights and development framework to a specific case study. This study adds to the literature and evidence base reaffirming the strong interconnection between health, human rights and development and the value of integrating their underlying concepts to ensure policies are not applied blindly and that the right-to-health is at the forefront of health services delivery.

## Supplementary information

**Additional file 1.** International and Regional Human Rights Conventions to which Mozambique is party.

## Data Availability

The datasets generated and analysed during the current study are not publicly available because providing access to individual de-identified data would exceed the scope of ethical approvals granted for this research. These are available from the corresponding author with permission from the Mozambican Ministry of Health on reasonable request.

## Abbreviations

ANC: Antenatal Care
CEDAW: Convention on the Elimination of All Forms of Discrimination Against Women
CHW: Community Health Worker
FGD: Focus Group Discussion
MNCH: Maternal Newborn and Child Health
MoH: Ministry of Health
PPH: Post-Partum Haemorrhage
SBA: Skilled Birth Attendant
SRH: Sexual and Reproductive Health
TBA: Traditional Birth Attendant
