# Supplementary material for: Misoprostol for the prevention of post-partum haemorrhage in Mozambique: an analysis of the interface between human rights, maternal health and development
Source: BMC Int Health Hum Rights. 2020 Apr 8;20:9. doi: 10.1186/s12914-020-00229-9 (PMC7140325; doi:10.1186/s12914-020-00229-9)
Supplement: Supplementary file 1 — Additional file 1. International and Regional Human Rights Conventions to which Mozambique is party. [file 12914_2020_229_MOESM1_ESM.docx]

| **Human Rights Instruments** | **Signed; ratified** | **Specific clause, or provision related to health and/or development** |
| --- | --- | --- |
| Optional Protocol to the African Charter on Human and Peoples’ Rights on the Rights of Women in Africa | 2005 (ratified) | Article 14 Health and Reproductive Rights |
| Solemn Declaration of Gender Equality in Africa | 2004 signed | Most points including 1. Promote gender specific economic, social and legal measures to combat the HIV/AIDS epidemic; 8; 9 and 10. Improve access to education, land and inheritance rights and reduce women’s workload (African Commission on Human and Peoples' Rights, 2018). |
| Convention against Torture and Other Cruel and Inhuman and Degrading Treatment or Punishment | 1999 | Acts of torture give rise to ill-health, whether physically or mentally and violate a person’s right to health. “By fulfilling the obligations under the Convention not to commit or allow acts of torture or other cruel, inhuman or degrading treatment or punishment that States parties indirectly also ensure an individual’s right to health” (World Health Organization, N/D, p. 2) |
| Southern African Development Community’s Declaration on Gender and Development | 1997 signed | The Declaration states that signatory governments are committed to: “Recognising, protecting and promoting the reproductive and sexual rights of women and girl child.” (Southern African Development Community (SADC), 2012, p. 4) |
| Convention on the Rights of the Child | 1994 | Article 24 recognises the rights of the child to the highest attainable standard of health and right of the mother to pre and post-natal care (UN General Assembly, 1989). |
| Convention on the Elimination of All Forms of Discrimination Against Women (CEDAW) | 1993 (ratified); in force 1997 | Article 12 of CEDAW makes specific mention to reproductive health rights; states have the responsibility to ensure that men and women have access to health services including family planning and women receive free pregnancy, post-natal and confinement health services, and information to decide on birth spacing (UN Women, 2009). |
| International Covenant on Civil and Political Rights | 1993 | Article 2 The right to non-discrimination  Article 6 The right to life  Article 23 The right to enter into marriage with free and full consent  Article 24 Right to birth registration |
| International Convention on the Elimination of All Forms of Racial Discrimination | 1983 | Article 5 (iv) “The right to public health, medical care, social security and social services” (United Nations Human Rights Office of the High Commissioner, 1996-2019) |
| International Covenant on Economic, Social and Cultural Rights (ICESCR) | Not a signatory | Article 12 and General Comment 14 the right to the highest attainable standard of health (United Nations Human Rights Office of the High Commissioner, N/D). |

**Additional file: 1. International and Regional Human Rights Conventions to which Mozambique is party**
